# Relationship between spinocranial angle and clinical outcomes after laminoplasty in patients with ossification of the posterior longitudinal ligament

**DOI:** 10.3389/fsurg.2022.1045085

**Published:** 2022-11-04

**Authors:** Zhen Liu, Zheng Wang, Peng Zhang, Wei Lin, De-Feng Liu, Xin Zhou, Ji-Hui Zheng

**Affiliations:** ^1^ Department of Spinal Surgery, Hebei Province Cangzhou Hospital of Integrated Traditional and Western Medicine, Cangzhou, China; ^2^ National Clinical Research Center for Geriatric Diseases, Xuanwu Hospital, Capital Medical University, Beijing, China

**Keywords:** ossification of the posterior longitudinal ligament, spinocranial angle, laminoplasty, clinical outcomes, t1 slope

## Abstract

**Background:**

The aims of this study were to identify the relationship between the spinocranial angle (SCA) and clinical outcomes and to explore whether the SCA is a suitable indicator to predict clinical outcomes for patients with ossification of the posterior longitudinal ligament (OPLL).

**Methods:**

Sixty-five patients with cervical OPLL who underwent laminoplasty with at least 24 months of follow-up were selected for the current study and were divided into two groups according to whether the SCA was greater than or less than the mean preoperative SCA. Sagittal alignment changes were compared between the groups. The Pearson correlation coefficient was applied to assess the relationship among sagittal parameters. Univariate and multiple linear regression analyses were applied to identify the relationship between the recovery rate (RR) and radiological parameters.

**Results:**

Patients were classified into two groups based on the mean value of preoperative SCA (85.1°). SCA was negatively correlated with T1 slope (T1s) and cervical lordosis (CL) and positively correlated with the C2–7 sagittal vertical axis (cSVA) (*p* < 0.001). Patients with lower SCA had larger T1s and CL preoperatively and at the follow-up (T1s: *p* < 0.001; CL: *p* < 0.001) and showed greater loss of cervical lordosis after laminoplasty (*p* < 0.001). However, no significant differences in the incidence of kyphosis, Japanese Orthopaedic Association (JOA) or RR were noted between the two groups. Although Pre-SCA, Pre-CL, F/U-CL and Pre-T1sCL were significantly associated with RR, these indicators were not associated with RR in the multivariate regression analysis.

**Conclusion:**

Patients with lower SCA tended to have higher T1s and CL before surgery and greater loss of cervical lordosis at the follow-up visit but still maintained a greater lordosis angle. Although preoperative SCA is significantly related to RR, the relationship is not sufficient to indicate that preoperative SCA can be used to predict clinical outcomes. Therefore, further research is needed to confirm the impact of SCA on clinical outcomes for OPLL.

## Introduction

Ossification of the posterior longitudinal ligament (OPLL) refers to a phenomenon of abnormal ossification of the ligament that is slow in the pathological process, and its specific pathogenesis is unclear. For cervical OPLL patients with severe clinical symptoms, anterior or posterior surgical intervention methods are currently used to facilitate decompression to relieve nerve compression and preserve nerve function. The scope of ossification lesions is often large and spans multiple segments. Patients with OPLL typically exhibit severe spinal cord compression accompanied by hypertrophy or ossification of the ligamentum flavum and spinal stenosis, which can easily cause nerve damage during anterior surgery. This notion led to increased interest in the use of laminoplasty in the treatment of cervical spine OPLL (1). Specifically, posterior spinal canal enlargement and laminoplasty achieved good prognostic effects during treatment (2, 3). Currently, cervical sagittal parameters are receiving increasing attention and are widely used to predict quality of life (4–7). Among them, the spinocranial angle (SCA), T1 slope (T1s) and C2–7 sagittal vertical axis (cSVA) are considered to be the three parameters that can better reflect sagittal balance and are also key research objects in the future (8). Regardless of whether it is T1s or cSVA, relevant studies on the evaluation of the sagittal alignment of cervical OPLL have been performed (9–11). However, although SCA, which is defined as the angle between a line from the sella turcica centre and C7 endplate and the C7 plateau line, has been reported to exhibit a significant correlation with many sagittal parameters (12), few studies have attempted to explore and correlate SCA with clinical results. Therefore, exploring the relationship between SCA and clinical outcomes is necessary.

The aim of our study was to explore the relationship between SCA and surgical effects after laminoplasty for cervical OPLL and to identify the significance of SCA as a predictor of clinical outcomes in patients with OPLL.

## Materials and methods

### Patient population

**Figure 1:**
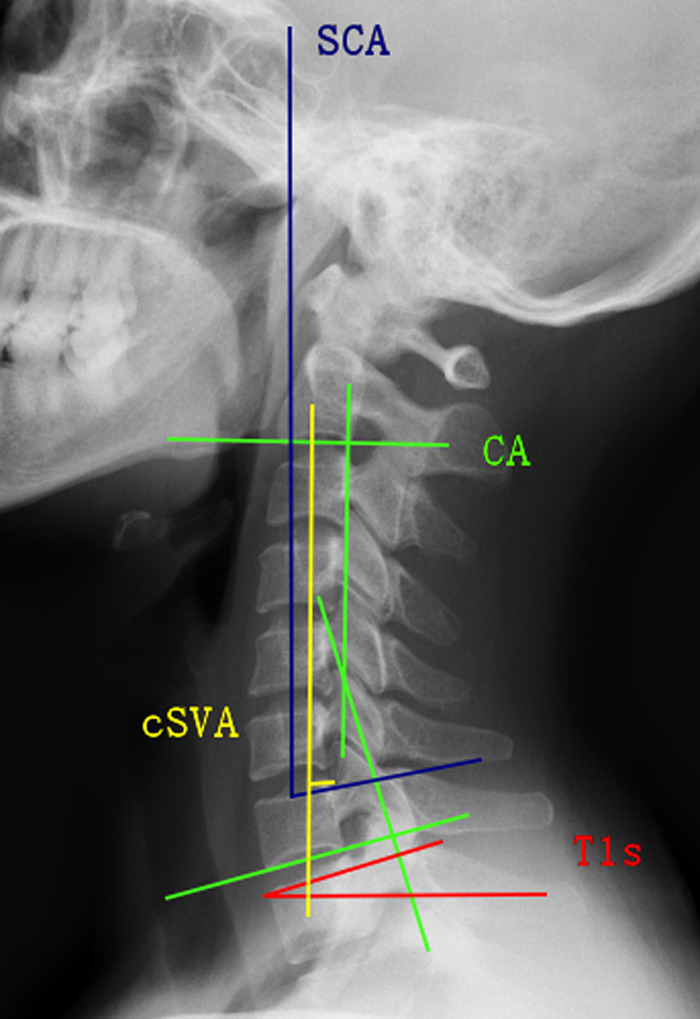
Spinocranial angle (SCA): the angle is defined as the angle between the C7 slope and the straight line joining the middle of the C7 end plate and the middle of the sella turcica. T1 slope (T1s): the angle between a horizontal line and the superior endplate of T1 or C7. C2–C7 lordosis (CL): the angle between the lower plate of C2 and the lower plate of C7. C2–C7 SVA (cSVA): the distance from the posterior, superior corner of C7 to the plumbline from the centroid of C2.

We retrospectively reviewed 65 consecutive patients (33 males and 32 females) with cervical OPLL who underwent laminoplasty between January 2010 and December 2016 in the Department of Spinal Surgery, the Third Hospital of Hebei Medical University (Figure 1). We included patients with (1) OPLL diagnosed by computed tomography; (2) completed radiographic and clinical data available; and (3) greater than 24 months of follow-up data. We excluded the following patients: (1) previous surgery involving the cervical spine; (2) cervical fractures, tumours, and metabolic disorders; (3) follow-up period less than 2 years; and (4) radiological parameters that were too unclear to measure. Health-related outcomes were evaluated preoperatively and at the follow-up period, including the Japanese Orthopaedic Association (JOA) (score 0–17) and recovery rate (RR) (postoperative score-preoperative score)/(17-preoperative score) × 100%).

### Radiographic analysis

**Figure 2:**
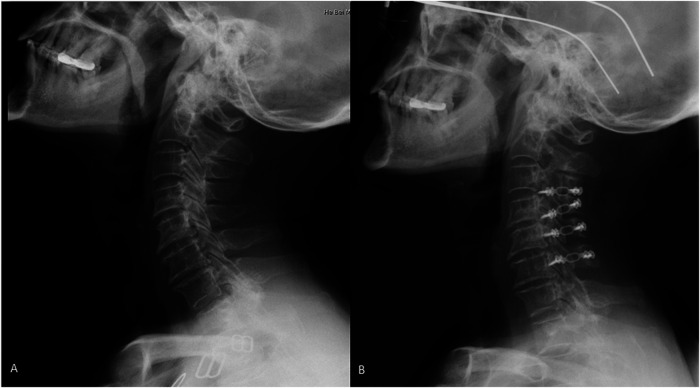
Posterior cervical surgery was performed to release the compression. Lateral x-ray of cervical spine was taken in a male patient with OPLL at preoperative and postoperative. **A** is preoperative, **B** is postoperative.

Lateral radiographs of the cervical spine were obtained preoperatively and at the 2-year follow-up. Radiological parameters included SCA, T1s, cervical lordosis (CL), cSVA, and T1sCL, which were measured as follows (Figure 2): (1) SCA is deﬁned as the angle defined between the C7 slope and the straight line joining the midpoint of the C7 end plate and the midpoint of the sella turcica. (2) T1s is deﬁned as the angle between the upper endplate of T1s and a horizontal line. (3) CL is deﬁned as the angle formed by the inferior end plates of C2 and C7. (4) cSVA is deﬁned as the horizontal distance from the posterior, superior corner of C7 vertebra to the plumbline from the centroid of C2 vertebra. (5) T1sCL is deﬁned as the angle that is calculated based on the T1 slope minus C2–C7 lordosis. Here, Δ represents the change of each sagittal parameter.

All of the patients had undergone posterior cervical singledoor laminoplasty. The surgeries were conducted by the same group of surgeons followed by the same procedure. The decompression and fixation surgeries were described briefly as follows: The patients were in the prone position after anesthesia was performed with close monitoring. After the skin, subcutaneous, and fascia were cut, bilateral paraspinal muscles were peeled off to expose the posterior structure of vertebral. The surgery only cut the muscle longitudinally, not horizontally. At the same time, the muscles that did not interfere with the surgery were left intact. The posterior vertebral plates were turned over and then fixed in a position where the spinal canal was enlarged. The decompression range of vertebral lamina is C3–C6.

### Statistical analysis

Data are revealed as the number of subjects in each group or the mean ± standard deviation and were calculated by SPSS (version 22.0; SPSS Inc., Chicago, IL, USA). Each independent variable was compared between the two groups using the independent-sample *t* test or Mann-Whitney *U* test and the *χ*^2^ test or Fisher's exact test. The Pearson correlation coefficient was applied to assess the relationships among preoperative SCA, preoperative T1s, preoperative CL, preoperative cSVA and preoperative T1sCL. Univariate and multiple linear regression analyses were applied to evaluate the relationship between RR and various sagittal parameters. Significance was noted at the *p* < 0.05 level.

## Results

### Comparison of patient backgrounds according to preoperative SCA

Sixty-five patients were selected for the current study and were divided into two groups according to the mean preoperative SCA (85.1°). Patient clinical features according to preoperative SCA are summarized in Table 1. The value of SCA varied from 67.9° to 83.9° in low-SCA group and from 85.3° to 105.6° in high-SCA group. No statistically significant differences in age, sex, type of OPLL, number of expanded laminae or incidence of diabetes mellitus were noted between the two groups. The prognostic indicators included F/U-JOA (low-SCA: 14.23 ± 1.09°, high-SCA: 13.94 ± 0.85°; *p* = 0.195) and RR (low-SCA: 59.32 ± 16.63°, high-SCA: 52.92 ± 15.66°; *p* = 0.067). None of the above indicators showed significant differences, except for F/U-JOA (low-SCA, *p* < 0.001; high-SCA, *p* < 0.001).

**Table 1:** Comparison of patient backgrounds according to preoperative SCA.

	Low-SCA group (lower half)	High-SCA group (upper half)	*p*-value
No. of patients	31	34	
Range of SCA (°)	67.9–83.9	85.3–105.6	
Age (year)	57.61 ± 8.29	59.62 ± 8.58	0.434^a^
Sex (male/female)	18/13	15/19	0.261^b^
Type of OPLL			0.674^b^
Continuous	6	9	
Segmental	10	12	
Mixed	15	13	
No. of expanded laminae	4.68 ± 1.23	4.53 ± 1.11	0.587^a^
incidence of diabetes mellitus	48.4% (15/31)	55.9% (19/34)	0.546^b^
JOA			
Pre	10.33 ± 1.64	10.15 ± 1.91	0.926^a^
F/U 2y	14.23 ± 1.09	13.94 ± 0.85	0.195^a^
Pre vs. F/U	<0.001^c^	<0.001^c^	
RR	59.32% ± 16.63%	52.92% ± 15.66%	0.067^a^

SCA, spino cranial angle; OPLL, ossiﬁcation of the posterior longitudinal ligament; Pre, preoperative; F/U, follow up; JOA, Japanese Orthopaedic Association; RRJOA, JOA recovery rate.

^a^ Mann-Whitney *U* test.

^b^ Chi-square test.

^c^ Wilcoxon Signed Ranks test.

### Comparison of radiologic parameters according to preoperative SCA

The values for and differences in radiological parameters between the two groups are summarized in Table 2. The following radiologic results were observed: Pre-T1s (27.59 ± 4.72° vs. 19.19 ± 3.90°, *p* < 0.001), F/U-T1s (25.10 ± 4.15° vs. 17.92 ± 4.30°, *p* < 0.001), Pre-CL (17.81 ± 4.60° vs. 8.90 ± 5.03°, *p* < 0.001), F/U-CL (12.62 ± 7.78° vs. 6.49 ± 4.22°, *p* < 0.001), Pre-cSVA (22.94 ± 9.16 vs. 23.46 ± 10.16 mm, *p* = 0.829), F/U-cSVA (26.27 ± 9.33 vs. 25.81 ± 9.48 mm, *p* = 0.845), Pre-T1sCL (9.78 ± 2.76° vs. 10.29 ± 5.39°, *p* = 0.895), and F/U-T1sCL (12.48 ± 7.55° vs. 11.43 ± 4.96°, *p* = 0.763). Only T1s and CL showed significant differences between the two groups both preoperatively and during the follow-up period.

**Table 2:** Comparison of radiologic and clinical parameters according to preoperative SCA.

	Low-SCA group (lower half)	High-SCA group (upper half)	*p*-value
**SCA (°)**			
Pre	76.51 ± 4.91	92.94 ± 5.72	<0.001^a^
F/U 2y	81.12 ± 7.63	94.63 ± 6.20	<0.001^a^
Pre vs. F/U	<0.001^b^	0.025^b^	
**T1s (°)**			
Pre	27.59 ± 4.72	19.19 ± 3.90	<0.001^c^
F/U 2y	25.10 ± 4.15	17.92 ± 4.30	<0.001^a^
Pre vs. F/U	<0.001^b^	0.042^d^	
**CA (°)**			
Pre	17.81 ± 4.60	8.90 ± 5.03	<0.001^c^
F/U 2y	12.62 ± 7.78	6.49 ± 4.22	<0.001^c^
Pre vs. F/U	<0.001^d^	<0.001^d^	
**cSVA (mm)**			
Pre	22.94 ± 9.16	23.46 ± 10.16	0.829^a^
F/U 2y	26.27 ± 9.33	25.81 ± 9.48	0.845^a^
Pre vs. F/U	0.010^b^	0.072^b^	
**T1sCA (°)**			
Pre	9.78 ± 2.76	10.29 ± 5.39	0.895^c^
F/U 2y	12.48 ± 7.55	11.43 ± 4.96	0.763^c^
Pre vs. F/U	0.026^d^	0.301^d^	

Pre, preoperative; F/U, follow up; SCA, spino cranial angle; T1s, T1-slope; CA, C2–7 lordosis angle; cSVA, C2–7 sagittal vertical axis; T1sCA, T1-slope minus C2–7 lordosis angle.

^a^ Independent *t*-test.

^b^ Paired *t*-test.

^c^ Mann-Whitney *U* test.

^d^ Wilcoxon Signed Ranks test.

### Comparison of sagittal alignment and clinical outcome changes according to preoperative SCA

Table 3 summarizes the changes in radiographic parameters and clinical efficacy. The mean values of ΔSCA and ΔCL were 4.61°, −5.18° in the low-SCA group and 1.69°, −2.41° in the high-SCA group, respectively, and all displayed significant differences (ΔSCA: *p* = 0.033, ΔCL: *p* < 0.001). However, the mean values of ΔT1s, ΔcSVA and ΔT1sCL were −2.49°, 3.33°, and 2.70° in the low-SCA group and −1.27°, 2.35°, and 1.14° in the high-SCA group, respectively. No significant differences were noted between the two groups (ΔT1s: *p* = 0.179, ΔcSVA: *p* = 0.948, ΔT1sCL: *p* = 0.222). Similarly, no significant difference occurred in ΔJOA between the two groups (*p* = 0.224).

**Table 3:** Comparison of sagittal alignment and clinical outcome changes according to preoperative SCA.

	Low-SCA group (lower half)	High-SCA group (upper half)	*p*-value
ΔSCA (°)	4.61 ± 6.47	1.69 ± 4.18	0.033^a^
ΔT1s (°)	−2.49 ± 3.34	−1.27 ± 3.84	0.179^a^
ΔCA (°)	−5.18 ± 5.20	−2.41 ± 2.36	<0.001^b^
ΔcSVA (mm)	3.33 ± 6.73	2.35 ± 7.35	0.948^b^
ΔT1sCA (°)	2.70 ± 6.59	1.14 ± 4.81	0.222^b^
ΔJOA	4.19 ± 1.60	3.79 ± 1.87	0.224^b^

SCA, spino cranial angle; T1s, T1-slope; CA, C2–7 lordosis angle; cSVA, C2–7 sagittal vertical axis; T1sCA, T1-slope minus C2–7 lordosis angle; JOA, Japanese Orthopaedic Association.

^a^ Independent *t*-test.

^b^ Mann-Whitney *U* test.

### Pearson correlations of cervical sagittal parameters

Table 4 demonstrates the Pearson correlations among preoperative sagittal parameters. Preoperative SCA was significantly correlated with T1s (*r* = −0.769, *p* < 0.001), CL (*r* = −0.856, *p* < 0.001) and preoperative cSVA (*r* = 0.430, *p* < 0.001). Preoperative T1s was positively correlated with preoperative CL (*r* = 0.768, *p* < 0.001). Preoperative CL was negatively correlated with preoperative cSVA (*r* = −0.395, *p* = 0.001) and T1sCL (*r* = −0.450, *p* < 0.001). Preoperative cSVA was positively correlated with preoperative T1sCL (*r* = 0.334, *p* = 0.007).

**Table 4:** Pearson correlations of preoperative cervical sagittal parameters.

	SCA	T1s	CA	cSVA
T1s	*r*	−0.769*			
	*p*	<0.001			
CA	*r*	−0.856*	0.768*		
	*p*	<0.001	<0.001		
cSVA	*r*	0.430*	−0.191	−0.395*	
	*p*	<0.001	0.128	0.001	
T1sCA	*r*	0.231	0.226	−0.450*	0.334*
	*p*	0.064	0.070	<0.001	0.007

SCA, spino cranial angle; T1s, T1-slope; CA, C2–7 lordosis angle; cSVA, C2–7 sagittal vertical axis; T1sCA, T1-slope minus C2–7 lordosis angle.

* Correlation is signiﬁcant at the 0.01 level (two-tailed).

### Univariate and multiple linear regression analysis of the relationship between RR and sagittal parameters

The results of univariate and multiple linear regression analyses are summarized in Tables 5, 6. Among all sagittal parameters, Pre-SCA, Pre-CL, F/U-CL and Pre-T1sCL were significantly related to RR (Pre-SCA: *r* = −0.247, *p* = 0.048; Pre-CL: *r *= 0.301, *p* = 0.015; F/U-CL: *r *= 0.247, *p* = 0.047; Pre-T1sCL: *r* = −0.334, *p* = 0.006). Unfortunately, the selected variables above showed no significant correlation with RR in multiple linear regression analysis.

**Table 5:** Unvariate analysis between RR and radiological parameters.

	*r*	*p*
Pre-SCA	−0.247*	0.048
F/U-SCA	−0.189	0.132
ΔSCA	0.108	0.391
Pre-T1s	0.089	0.481
F/U-T1s	0.195	0.119
ΔT1s	0.151	0.230
Pre-CA	0.301*	0.015
F/U-CA	0.247*	0.047
ΔCA	−0.067	0.595
Pre-cSVA	0.036	0.774
F/U-cSVA	0.065	0.609
ΔcSVA	0.036	0.776
Pre-T1sCA	−0.334**	0.006
F/U-T1sCA	−0.098	0.440
ΔT1sCA	0.145	0.250

Pre, preoperative; F/U, follow up; SCA, spino cranial angle; T1s, T1-slope; CA, C2–7 lordosis angle; cSVA, C2–7 sagittal vertical axis; T1sCA, T1-slope minus C2–7 lordosis angle; RR, recovery rate.

* Correlation is signiﬁcant at the 0.05 level (two-tailed).

** Correlation is signiﬁcant at the 0.01 level (two-tailed).

**Table 6:** Multivariate analysis of factors associated with RR.

Parameters	*B*	Se	Beta	*t*	*p*
Pre-SCA (°)	−0.002	0.004	−0.114	−0.459	0.648
Pre-CA (°)	0.002	0.008	0.084	0.257	0.798
F/U-CA (°)	<0.001	0.005	−0.003	−0.016	0.987
Pre-T1sCA (°)	−0.010	0.005	−0.272	−1.911	0.061

PRE, preoperative; F/U, follow up; SCA, spino cranial angle; CA, C2–7 lordosis angle; T1sCA, T1s minus CA; RR, recovery rate.

## Discussion

Recently, the significance of cervical alignment balance based on sagittal parameters has been gradually realized (4, 5). Cervical sagittal parameters exhibit a close correlation with quality of life (6, 7). Poor cervical equilibrium after the posterior approach is widely recognized as an important influencing factor leading to a decline in quality of life (6, 13). Among numerous sagittal parameters, three parameters stand out: SCA, T1s and cSVA. Previous reports have evaluated sagittal balance by SCA, which fluctuates within a certain range (83° ± 9°) under normal conditions and is significantly correlated with T1s and CL (12). Although the essential sagittal parameter of SCA is being gradually recognized as an important factor, there are limited reports on the role of SCA in sagittal balance. In addition, whether SCA has the ability to predict changes in the sagittal sequence and clinical results, such as T1s and cSVA, remains unclear (10). Moreover, whether the degree of cervical sagittal balance damage after laminoplasty is associated with preoperative sagittal parameters remains controversial (14, 15). In our study, patients with a lower SCA who underwent laminoplasty had more changes in sagittal parameters, such as an increase in the SCA and the loss of CL. Simultaneously, preoperative SCA showed a negative correlation with T1s and CL. Moreover, T1s was significantly positively correlated with CL, which is consistent with previous reports that higher T1s tend to be accompanied by higher CL (5, 15). Studies have shown that patients with higher T1s may have higher CL, and greater effort is required to maintain cervical alignment balance (14, 15). Our research results seem to apply this hypothetical conclusion to SCA as well, and the results can be generalized to OPLL. In the present study, compared with the preoperative period, all the sagittal parameters involved in the low-SCA group were significantly changed during the follow-up period. However, only T1s and CL were significantly changed in the high-SCA group during the follow-up period, and the changes in SCA and CL in the high-SCA group were significantly smaller than those in the low-SCA group. However, for clinical results, such as JOA and RR, no significant difference was noted between the two groups. This finding may explain why patients with lower SCAs are more susceptible altered sagittal balance after surgery. Sagittal malalignment has been confirmed to be closely associated with a decline in health status, and rational equilibrium could contribute to maintaining posture and ameliorating quality of life (16–18). Moreover, it remains controversial whether cervical kyphosis is associated with RR in patients after laminoplasty (19, 20). Therefore, we hypothesized that SCA might also be associated with clinical prognosis, so we established a univariate regression analysis model to try to correlate various sagittal parameters with RR. Although Pre-SCA, Pre-CL, F/U-CL and Pre-T1sCA were significantly associated with RR, these indicators were not associated with RR in the multivariate regression analysis model. Moreover, neither JOA nor RR significantly differed between the two groups. Therefore, SCA does not seem to be a predictor of clinical outcomes. However, we consider that each specific disease should have a corresponding range of appropriate sagittal parameters, and the relevant conclusions are also applicable to different diseases. Therefore, SCA may not be an appropriate parameter to predict prognostic efficacy in OPLL. Although no significant difference in clinical outcomes was noted between the two groups, the alterations in alignment deserve our attention. Although the loss of cervical lordosis in the low-SCA group was greater than that in the high-SCA group, cervical lordosis could still be maintained. In addition, cervical lordosis was significantly greater than that in the high-SCA group, whereas the change in SCA in the low-SCA group was also larger than that in the high-SCA group. These results indicate that the smaller the SCA and the greater the CL, the more prominent the changes in sagittal balance after laminoplasty. However, the relationship between the surgical effect and SCA changes remains uncertain. No significant differences in cSVA, T1sCL or the incidence of kyphosis were noted between the two groups at the preoperative and follow-up visits, suggesting that patients may be compensated by the global alignment of the spine. Therefore, we believe that cervical alignment is easier to maintain in normal order for patients who can effectively compensate.

Our study has several significant limitations. The first is related to retrospective design. Moreover, the average follow-up time was 28 months, which is too short. In addition, the sample sizes were relatively small. Second, no comprehensive evaluation of clinical and functional results was performed, and only JOA and its RR were statistically evaluated. Third, sagittal x-ray examination of the global spine was not performed, so the relationship between SCA and global sagittal balance could not be further determined. However, despite these limitations, our study is valuable for understanding the relationship between SCA and clinical outcomes after posterior cervical surgery in patients with cervical OPLL.

## Conclusion

Our study demonstrated that compared with the high-SCA group, patients with lower SCA tended to have higher T1s and CL before laminoplasty and greater loss of cervical lordosis at the follow-up visit but still maintained a greater lordosis angle. Although preoperative SCA is significantly related to RR, it is not sufficient to indicate that preoperative SCA can be used to predict clinical outcomes. Therefore, further research is needed to confirm the impact of SCA on clinical outcomes for OPLL.

## Data Availability

The raw data supporting the conclusions of this article will be made available by the authors, without undue reservation.
